# Body Mass Index and Outcomes in HR+/HER2− Metastatic Breast Cancer Treated with Palbociclib: Insights from a National Real-World Study

**DOI:** 10.3390/cancers18091379

**Published:** 2026-04-26

**Authors:** Larisa Maria Badau, Paul Epure, Madalin-Marius Margan, Roxana Margan, Andrei Dorin Ciocoiu, Cristina Marinela Oprean, Brigitha Vlaicu

**Affiliations:** 1 Doctoral School in Medicine, “Victor Babes” University of Medicine and Pharmacy, Eftimie Murgu Square No. 2, 300041 Timisoara, Romania; larisa.badau@umft.ro (L.M.B.); andrei.ciocoiu@umft.ro (A.D.C.); 2 Department of Oncology, ONCOHELP Hospital Timisoara, Ciprian Porumbescu Street, No. 59, 300239 Timisoara, Romania; cristina.oprean@umft.ro; 3 Discipline of Hygiene, “Victor Babes” University of Medicine and Pharmacy, Eftimie Murgu Square No. 2, 300041 Timisoara, Romania; roxana.margan@umft.ro (R.M.); vlaicu@umft.ro (B.V.); 4 ”Pius Brinzeu” County Emergency Hospital, 300723 Timisoara, Romania; epurepaul1297@gmail.com; 5 Department of Public Health and Sanitary Management, “Victor Babes” University of Medicine and Pharmacy, Eftimie Murgu Square No. 2, 300041 Timisoara, Romania; 6 Center for Studies in Preventive Medicine, “Victor Babes” University of Medicine and Pharmacy, 300041 Timisoara, Romania; 7 Department of Oncology, City Clinical Emergency Hospital of Timisoara, Victor Babes Blvd. No. 22, 300595 Timisoara, Romania; 8 Department of Oncology, ONCOMED Outpatient Unit Timisoara, Ciprian Porumbescu Street, No. 59, 300239 Timisoara, Romania

**Keywords:** metastatic breast cancer, body mass index, palbociclib, CDK4/6 inhibitors, progression-free survival, overall survival, real-world

## Abstract

**Background/Objectives**: The prognostic and predictive role of BMI in patients with HR+/HER2− MBC remains controversial, particularly in the era of CDK4/6 inhibitors. This study aimed to evaluate the association between baseline BMI and clinical outcomes in patients treated with palbociclib in a real-world setting. **Methods**: We conducted a multicenter retrospective observational cohort study including 326 patients with HR+/HER2− MBC treated with palbociclib in combination with endocrine therapy across six oncology centers in Romania. Only patients who received palbociclib for at least three months were included. Patients were stratified according to BMI into <25 kg/m^2^ and ≥25 kg/m^2^ groups. PFS and OS were the primary endpoints, while ORR and CBR were secondary endpoints. **Results**: Among the 326 patients, 66.56% were classified as overweight or obese (BMI ≥ 25 kg/m^2^). Median PFS was 23.66 months in the BMI < 25 group and 26.78 months in the BMI ≥ 25 group, with no statistically significant difference (HR 0.86; 95% CI 0.62–1.20; *p* = 0.373). Median OS was not reached in the BMI < 25 group and was 43.73 months in the BMI ≥ 25 group, also without a significant difference (HR 0.82; 95% CI 0.52–1.30; *p* = 0.397). ORR (29.07% vs. 28.89%) and CBR (90.70% vs. 88.33%) were comparable between BMI groups. In multivariate analysis, liver metastases and brain metastases were independently associated with worse outcomes, whereas BMI was not an independent prognostic factor. **Conclusions**: In this selected real-world cohort of patients with HR+/HER2− MBC who tolerated at least three months of palbociclib, baseline BMI was not associated with treatment response, PFS, or OS. While clinically informative, these results should not be interpreted as definitive evidence that body weight has no influence on palbociclib efficacy, given the methodological constraints of the analysis. BMI alone may be insufficient to capture the complex interplay between body composition and treatment outcomes, highlighting the need for more refined biomarkers of body composition in this setting.

## Simple Summary

Body weight and obesity are increasingly recognized as factors that may influence cancer outcomes; however, their role in metastatic breast cancer remains incompletely defined. In this study, we evaluated real-world data from patients treated with palbociclib, a widely used therapy for hormone receptor-positive metastatic breast cancer, to determine whether BMI affects treatment response and survival. We found that BMI was not associated with progression-free survival, overall survival, or response to treatment. Although informative, these findings should not be interpreted as definitive evidence that body weight has no influence on palbociclib efficacy. However, BMI alone may not accurately reflect body composition, and more precise measures are needed. Our findings contribute to a better understanding of how patient-related factors influence treatment outcomes and support a more individualized approach to cancer care.

## 1. Introduction

Body mass index (BMI), a widely used indicator of nutritional status, has emerged as a growing focus of interest in oncologic research [1]. BMI, calculated as weight in kilograms divided by height in meters squared, is the most commonly used measure to identify excess adiposity and estimate its severity [2]. According to the World Health Organization (WHO), obesity is defined as a BMI of ≥30 kg/m^2^ and has been consistently associated with an increased risk of several cancers, including breast cancer (BC) [3].

Obesity has become a major global health concern and has been largely attributed to sedentary behavior, hypercaloric diets, and a complex interplay of environmental and socioeconomic factors associated with the modern Western lifestyle [4]. Between 1990 and 2022, the global age-standardized prevalence of obesity in women increased from 4.8% to 18.5%, affecting approximately 504 million women worldwide [4].

BC remains the most common malignancy in women, and its incidence has been increasing across Europe, which is believed to be partly associated with the rising prevalence of obesity [5]. Given this context, obesity and weight-related parameters have been increasingly investigated as potential prognostic and predictive factors for treatment outcomes and survival in BC [6]. However, BMI has several well-recognized limitations, including its inability to distinguish between fat mass and lean mass or to assess fat distribution, which may result in under- or overestimation of obesity [7]. Despite these limitations, BMI remains widely used because of its simplicity, consistency, and applicability in large-scale epidemiological research [2].

The relationship between BMI and BC outcomes is complex and influenced by multiple factors, including disease stage, tumor biological subtype and treatment modality. In early-stage breast cancer (EBC), particularly in hormone receptor-positive (HR+) subtypes, obesity has been consistently associated with poorer prognosis in both premenopausal and postmenopausal women [8]. This effect is largely attributed to increased estrogen production by adipose tissue, which binds to estrogen receptors on HR+ BC cells, thereby promoting tumor growth and enhanced cellular proliferation [9]. Accordingly, the National Comprehensive Cancer Network (NCCN) guidelines recommend maintaining a BMI below 25 kg/m^2^ in patients with BC [10].

In contrast, in the metastatic setting, the role of BMI on prognosis remains controversial. Although certain studies have associated higher BMI with worse outcomes in metastatic breast cancer (MBC) [11], others have failed to demonstrate a significant association [12,13,14]. This phenomenon, often referred to as the “obesity paradox,” has been described in several malignancies, including lung cancer, renal cell carcinoma, and melanoma [15]. Obesity is widely recognized as being strongly linked to cancer progression through chronic inflammation and hormonal alterations mediated by adipocyte-derived factors, such as leptin [16].

BC is a biologically heterogeneous disease, with approximately 70% of cases classified as HR+/human epidermal growth factor receptor 2–negative (HER2−) [17]. Given the high prevalence of this subtype, identifying its prognostic or predictive factors remains a critical priority in clinical practice.

Following its approval in 2015, palbociclib and subsequently other cyclin-dependent kinase 4/6 (CDK4/6) inhibitors have become a cornerstone of therapy for HR+/HER2− MBC. Large phase III clinical trials such as PALOMA, MONALEESA, MONARCH, and DAWNA have demonstrated that CDK4/6 inhibitors including palbociclib, ribociclib, abemaciclib and dalpiciclib, significantly improve progression-free survival (PFS) and overall survival (OS) [18].

However, evidence regarding the association between BMI and clinical outcomes in HR+/HER2− MBC treated with CDK4/6 inhibitors remains limited and inconsistent [19,20,21,22]. Given the involvement of CDK4/6 inhibitors in the regulation of lipogenesis and their potential interaction with obesity-related metabolic pathways, further investigation is warranted to clarify how obesity may influence treatment response and prognosis in this patient population [23].

Therefore, we conducted a national, multicenter real-world study to investigate the impact of baseline BMI on treatment response and survival outcomes in patients with HR+/HER2− MBC treated with palbociclib in first- and second-line settings.

## 2. Materials and Methods

### 2.1. Study Design

A multicenter observational cohort study was conducted in six oncology centers across Romania. Real-world data were retrospectively collected between November 2021 and January 2025, with follow-up data available until January 2025. Survival follow-up was extended up to approximately 45 months. Eligible participants were adult women (≥18 years) with de novo or recurrent HR+/HER2− MBC who received palbociclib as a first- or second-line therapy. The initial study database included all consecutive patients treated with palbociclib in the participating centers during the data collection period. The original study cohort included 344 patients who met the predefined eligibility criteria, including a minimum of 3 months of palbociclib treatment, allowing adequate treatment exposure for outcome assessment, and the absence of severe symptomatic visceral disease, as specified in the study protocol. This criterion may, however, have introduced selection bias by excluding patients who experienced rapid disease progression, early death, or early treatment discontinuation due to toxicity or clinical deterioration. Hormone receptor positivity was defined as immunohistochemical estrogen receptor and/or progesterone receptor expression > 1% based on local laboratory results. HER2− status was defined as an immunohistochemistry score of 0 or 1+, or a score of 2+ with negative amplification by fluorescence in situ hybridization (FISH)/in situ hybridization (ISH).

In this secondary analysis, baseline BMI was evaluated as the exposure of interest within the previously described observational dataset to assess its association with clinical outcomes in patients treated with palbociclib.

For the purpose of the present analysis, patients without available baseline height and weight data were excluded, precluding BMI calculation. A total of 18 patients were excluded for this reason, resulting in a final analytical cohort of 326 patients. The final analytical cohort, therefore, represents a selected population with available baseline anthropometric data and sufficient treatment exposure.

Further details of the study design and methodology have been previously reported in the published study protocol [24].

The study protocol (PALBO01/2021, version number 3/19 December 2022) was approved by the institutional review boards of the participating centers and by the National Ethical Committee (approval No. 28 SNI, 27 October 2022).

### 2.2. Data Collection

Patients with missing BMI data were excluded from the analysis, while for other variables, analyses were performed using available data without imputation. The proportion of missing data was low and was not expected to have a significant impact on the results. Clinicopathological characteristics and treatment-related variables were retrospectively collected from institutional records and entered into electronic case report forms, with data from external sources incorporated into the electronic data capture system for final analysis. Missing data were handled using a complete-case analysis approach.

Baseline BMI was calculated for all patients as body weight (kg) divided by height squared (m^2^). Height and weight values used for BMI calculation were obtained from medical records at the time of palbociclib initiation (baseline). Patients were classified according to the WHO criteria [25]: underweight (<18.5 kg/m^2^), normal weight (18.5–24.9 kg/m^2^), overweight (25.0–29.9 kg/m^2^), and obese (≥30.0 kg/m^2^). The underweight and normal-weight patients were grouped together (BMI < 25 kg/m^2^), while overweight and obese patients were combined into a second category (BMI ≥ 25 kg/m^2^). Furthermore, subgroup analyses were performed separately in patients receiving palbociclib as first-line therapy (group 1) and second-line therapy (group 2).

### 2.3. Objectives and Endpoints

Our study aimed to compare the effectiveness and treatment response to palbociclib in combination with endocrine therapy administered as first- or second-line treatment according to BMI status in patients with HR+/HER2− MBC. The primary endpoints were PFS and OS, while secondary endpoints included treatment response.

Treatment effectiveness was assessed using PFS and OS as clinical outcome measures. PFS was defined as the time from treatment initiation to disease progression or death, whichever occurred first, while OS was defined as the time from treatment initiation to death from any cause. The survival follow-up was conducted at 12, 24, and 30 months. Patients were followed until disease progression, death, or last clinical contact, with a maximum follow-up of approximately 45 months.

Treatment response was assessed using overall response rate (ORR) and clinical benefit rate (CBR). ORR was defined as the proportion of patients with complete response (CR) or partial response (PR). CBR was defined as the proportion of patients achieving CR + PR + stable disease (SD) after 6 months of therapy. In the present cohort, all SD cases met this duration criterion.

Patients without response data or non-measurable disease (isolated metastasis to bone, pleura, or skin) were considered not evaluable. Tumor response was assessed according to Response Evaluation Criteria in Solid Tumors (RECIST) version 1.1 criteria based on retrospectively collected computed tomography (CT) scans performed at approximately three-month intervals during follow-up.

In addition, factors associated with PFS and OS were explored.

### 2.4. Statistical Analysis

Descriptive statistical analyses were performed to summarize patient demographic, clinicopathological and treatment characteristics stratified by BMI categories for both categorical and continuous variables. Continuous variables were presented as mean ± standard deviation or median (interquartile range), depending on their distribution, while categorical variables were presented as counts and percentages. Comparisons between BMI groups were performed using the chi-square test or Fisher’s exact test for categorical variables, and the Mann–Whitney U test for continuous variables, as appropriate.

PFS and OS were calculated from the date of palbociclib initiation to the date of disease progression and the last follow-up or death from any cause, respectively. Patients without an event were censored at the date of last follow-up. Survival curves were analyzed using the Kaplan–Meier method and compared between groups using the log-rank test. Kaplan–Meier curves were generated using Python (version 3.14.0; Python Software Foundation, Wilmington, DE, USA). Cox proportional hazards regression analyses were performed to identify factors associated with PFS and OS in both univariate and multivariate models, with results reported as hazard ratios (HR) and 95% confidence intervals (CI). The proportional hazards assumption was assessed graphically and statistically, and no major violations were identified. Variables were selected for inclusion in the multivariate models based on statistical significance in univariate results and clinical relevance. Treatment response was compared between BMI groups using logistic regression analysis, and the results were reported as odds ratios (ORs) with 95% CIs. All tests were two-sided, and statistical significance was defined as a *p*-value < 0.05.

Given the multicenter design, potential heterogeneity between centers was considered. However, due to the relatively homogeneous treatment approach and the absence of significant center-level variability in baseline characteristics, center of inclusion was not included as a covariate in the final models. BMI was analyzed as a categorical variable using a predefined threshold (≥25 kg/m^2^) to reflect clinically relevant categories and to ensure adequate group sizes. We acknowledge that alternative approaches, including modeling BMI as a continuous variable or using different thresholds, may provide additional insights and should be explored in future studies.

All statistical analyses were performed using R software (version 4.4.3; R Foundation for Statistical Computing, Vienna, Austria).

## 3. Results

### 3.1. Patient Characteristics

This analysis included 326 female patients with HR+/HER2− MBC with sufficient treatment exposure for response and survival assessment. Patients were stratified according to BMI into two groups: <25 kg/m^2^ (*n* = 109) and ≥25 kg/m^2^ (*n* = 217). Baseline characteristics were generally well balanced between BMI groups, with the exception of age at diagnosis and hypertension, with no significant differences observed in other demographic, clinical, or treatment characteristics (Table 1). The median age of the study population was 60 years (range: 49–67). Patients with higher BMI were significantly older compared to those with lower BMI (61 vs. 54 years; *p* = 0.039). Although not statistically significant, postmenopausal status was more frequent in patients with BMI ≥ 25 kg/m^2^ than in those with BMI < 25 kg/m^2^ (84.79% vs. 77.06%; *p* = 0.085).

The presence of comorbidities such as hypertension was more prevalent in the higher BMI group (42.40% vs. 27.52%; *p* = 0.009), while diabetes was numerically more frequent but did not reach statistical significance (*p* = 0.227). No significant differences were observed between BMI groups regarding tumor characteristics (histology, grade, luminal subtype, Ki-67, HER2 status). The majority of tumors were invasive ductal carcinoma (57.67%), luminal B (60.74%) and HER2-negative (52.92%).

A total of 139 patients (42.6%) had previously received neo/adjuvant endocrine therapy, while 108 patients (33.1%) had been treated with neo/adjuvant chemotherapy. Approximately half of the patients (56.44%) had de novo metastatic disease, and 54.29% had visceral involvement. A total of 140 patients (42.94%) presented with a single metastatic site.

Regarding treatment line, the majority of patients were treated with palbociclib in the first-line setting (229 patients, 70.25%). Most patients received an aromatase inhibitor (AI) as the endocrine partner (232 patients, 71.2%), with a higher distribution observed in the BMI ≥ 25 kg/m^2^ group (*p* = 0.711).

### 3.2. Tumor Response to Palbociclib According to BMI

Among the 326 patients included in the study, 266 (81.6%) were evaluable for tumor response, including 86 (78.9%) in the BMI < 25 kg/m^2^ group and 180 (83.0%) in the BMI ≥ 25 kg/m^2^ group (Table 2). No significant differences were observed between BMI groups in terms of ORR, BOR or CBR. Importantly, no cases of SD with a duration of less than 6 months were observed among evaluable patients. Therefore, all SD cases fulfilled the ≥6 months criterion and were included in the calculation of the CBR.

The ORR was 29.07% in the BMI < 25 group and 28.89% in the BMI ≥ 25 group (odds ratio [OR], 1.03; 95% confidence interval [CI], 0.58–1.86; *p* = 0.918). Similarly, CBR was comparable between groups (90.70% vs. 88.33%; OR, 0.77; 95% CI, 0.30–1.79; *p* = 0.551).

### 3.3. Progression-Free Survival Outcome

At the time of analysis, the maximum follow-up was approximately 45 months.

**Figure 1:**
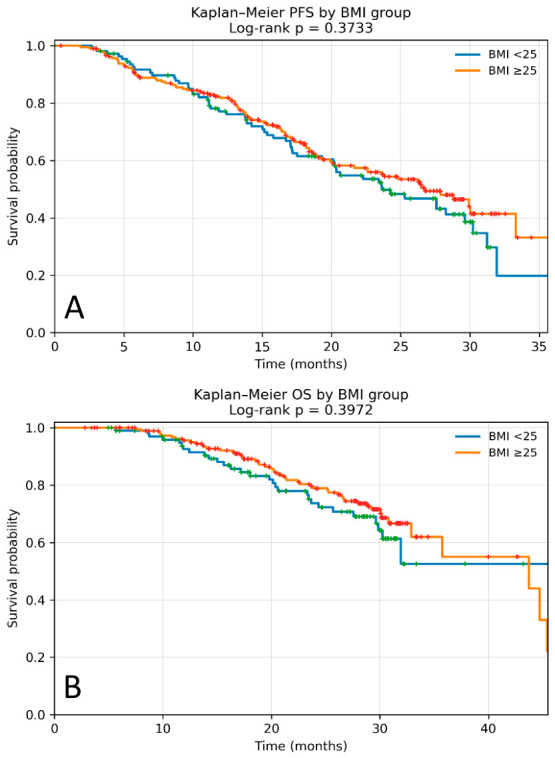
Kaplan–Meier curves according to BMI (<25 vs. ≥25 kg/m^2^) in the overall cohort of patients with HR+/HER2− MBC treated with palbociclib. (**A**) Progression-free survival (PFS). Median PFS was 23.66 months in the BMI < 25 kg/m^2^ group and 26.78 months in the BMI ≥ 25 kg/m^2^ group, with no statistically significant difference between groups (log-rank *p* = 0.373). (**B**) Overall survival (OS). Median OS was not reached in the BMI < 25 kg/m^2^ group and was 43.73 months in the BMI ≥ 25 kg/m^2^ group, with no statistically significant difference (log-rank *p* = 0.397). Censored cases are indicated by plus symbols.

In the overall cohort, the median PFS was 26.45 months (95% CI 22.28–29.93). Median PFS was 23.66 months in patients with BMI < 25 kg/m^2^ and 26.78 months in those with BMI ≥ 25 kg/m^2^. Although a numerically longer PFS was observed in patients with higher BMI, this difference was not statistically significant (HR 0.86, 95% CI 0.62–1.20; *p* = 0.373) (Figure 1A).

In the univariate analysis, luminal B subtype, liver metastases, visceral metastatic involvement, a higher number of metastatic sites, and second-line palbociclib treatment were associated with shorter PFS. Ki-67 < 20% was associated with longer PFS compared with Ki-67 ≥ 20% (HR 0.77; 95% CI 0.61–0.97; *p* = 0.025) (Table 3).

In the multivariate Cox regression analysis, only liver metastases remained an independent predictor of shorter PFS (HR 1.59; 95% CI 1.19–2.14; *p* = 0.002). None of the other clinicopathological variables retained statistical significance. BMI showed no significant association with PFS in either univariate or multivariate analysis (Table 3).

**Figure 2:**
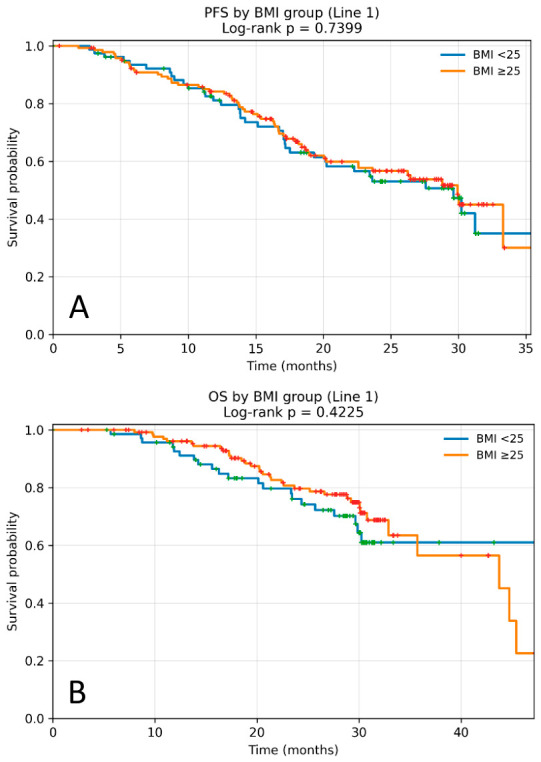
Kaplan–Meier curves according to BMI (<25 vs. ≥25 kg/m^2^) in the subgroup of patients receiving first-line palbociclib. (**A**) Progression-free survival (PFS). Median PFS was 29.63 months in the BMI < 25 kg/m^2^ group and 29.93 months in the BMI ≥ 25 kg/m^2^ group, with no statistically significant difference between groups (log-rank *p* = 0.739). (**B**) Overall survival (OS). Median OS was not reached in the BMI < 25 kg/m^2^ group and was 43.73 months (95% CI 32.80–52.40) in the BMI ≥ 25 kg/m^2^ group, with no statistically significant difference (log-rank *p* = 0.422). Censored cases are indicated by plus symbols.

Subgroup analyses according to treatment line showed consistent results. In the patients receiving first-line palbociclib (*n*= 229), median PFS was 29.63 months (95% CI 24.30–33.80). Median PFS was similar between BMI groups (29.63 vs. 29.93 months; HR 0.93; 95% CI 0.62–1.41; *p* = 0.739) with no statistically significant difference (Figure 2A).

**Figure 3:**
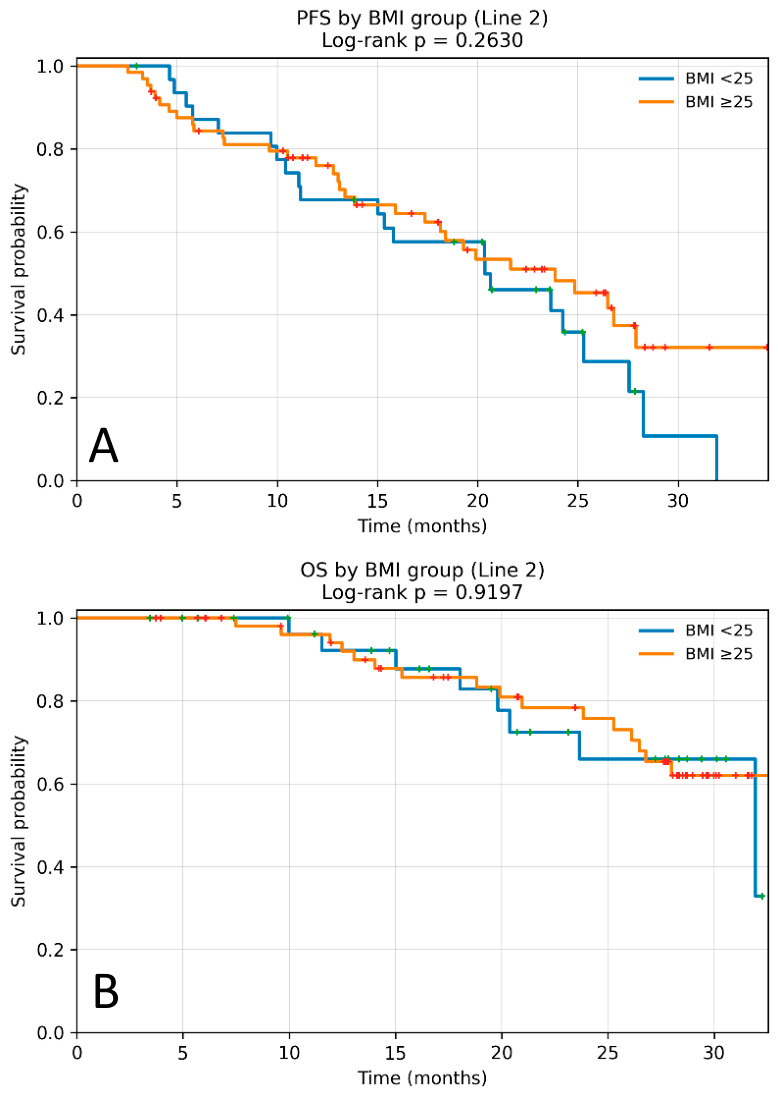
Kaplan–Meier curves according to BMI (<25 vs. ≥25 kg/m^2^) in the subgroup of patients receiving second-line palbociclib. (**A**) Progression-free survival (PFS). Median PFS was 20.37 months in the BMI < 25 kg/m^2^ group and 23.85 months in the BMI ≥ 25 kg/m^2^ group, with no statistically significant difference between groups (log-rank *p* = 0.263). (**B**) Overall survival (OS). Median OS was 31.93 months in the BMI < 25 kg/m^2^ group and was not reached in the BMI ≥ 25 kg/m^2^ group, with no statistically significant difference (log-rank *p* = 0.919). Censored cases are indicated by plus symbols.

In the second-line setting (*n*= 97), median PFS was 21.65 months (95% CI 17.90–26.80). Median PFS did not differ significantly between BMI groups (20.37 vs. 23.85 months; HR 0.73; 95% CI 0.40–1.34; *p* = 0.263) (Figure 3A).

### 3.4. Overall Survival Outcome

In the overall cohort, the median OS was 43.73 months (95% CI 32.89–52.20). Median OS was not reached in patients with BMI < 25 kg/m^2^ and was 43.73 months (95% CI 32.89–45.40) in those with BMI ≥ 25 kg/m^2^, with no statistically significant difference between groups (HR 0.82; 95% CI 0.52–1.30; *p* = 0.397) (Figure 1B).

In the univariate analysis, other histologic types, higher tumor grade, luminal B subtype, the presence of brain and liver metastases, visceral metastatic involvement, and a higher number of metastatic sites were associated with poorer OS. A lower Ki-67 index (<20%) was associated with improved OS (Table 4).

In the multivariate analysis, two variables remained independent predictors of OS. Liver metastases were associated with significantly shorter OS (HR 2.33; 95% CI 1.40–3.88; *p* = 0.001), while brain metastases also independently predicted poorer outcomes (HR 2.78; 95% CI 1.16–6.66; *p* = 0.022). BMI showed no significant association with OS in either univariate or multivariate analysis (Table 4).

In subgroup analyses stratified by treatment line, BMI was not significantly associated with OS. In the subgroup of patients receiving first-line palbociclib, median OS was not reached in the BMI < 25 group, whereas the patients with BMI ≥ 25 kg/m^2^ had a median OS of 43.73 months (95% CI 32.80–52.40), with no statistically significant difference between BMI groups (HR 0.74; 95% CI 0.43–1.29; *p* = 0.422) (Figure 2B).

In the second-line setting, median OS was not reached in the BMI ≥ 25 kg/m^2^, whereas the patients with BMI < 25 kg/m^2^ had a median OS of 31.93 months (95% CI 19.80–47.30). BMI was not significantly associated with OS in this subgroup (HR 0.96; 95% CI 0.40–2.26; *p* = 0.919) (Figure 3B).

## 4. Discussion

The potential role of BMI as a predictor of treatment response and survival in HR+/HER2− MBC remains a matter of debate, particularly in the era of CDK4/6 inhibitors. In this multicenter real-world study including 326 patients, we evaluated the association between BMI and the therapeutic efficacy of palbociclib in combination with endocrine therapy. Notably, we observed a high baseline prevalence of overweight and obesity (66.56%), reflecting real-world patient populations and supporting the clinical relevance of exploring BMI as a potential prognostic factor. The study comprised several key components, including the characterization of the patient population treated with palbociclib according to BMI categories, the evaluation of PFS and OS across BMI subgroups and treatment lines, as well as the assessment of response rates. No significant association was observed between BMI and clinical outcomes or response rates, and this finding was consistent across treatment lines. BMI also did not emerge as an independent prognostic factor in multivariate analyses. Among the evaluated variables, liver and brain metastases were independently associated with worse clinical outcomes, while other factors did not retain statistical significance.

The available evidence regarding the prognostic and predictive role of BMI in patients treated with CDK4/6 inhibitors remains heterogeneous. While some retrospective studies have reported a potential association between higher BMI and improved clinical outcomes [20,21,26], others have not confirmed a statistically significant relationship [13,22,27,28]. A retrospective cohort study evaluating CT-derived body composition parameters demonstrated that BMI and adiposity indices were not prognostic for PFS, whereas skeletal muscle mass (sarcopenia) emerged as a significant determinant of outcomes [27]. In this context, our real-world findings are aligned with those of our previously published systematic review, which concluded that BMI alone is insufficient to serve as a reliable prognostic or predictive biomarker in patients receiving CDK4/6 inhibitors [29].

The apparent trend toward improved PFS among patients with higher BMI may be partially explained by pharmacokinetic differences and reduced treatment-related toxicity. Patients with lower BMI may experience higher plasma drug concentrations with fixed dosing, potentially leading to increased hematologic toxicity and treatment modifications [30], whereas a larger volume of distribution in patients with higher BMI may result in lower drug exposure and fewer adverse events [31]. These mechanisms have been suggested in previous studies, including subgroup analyses from clinical trials such as PALOMA-3 and in Asian populations, who generally have lower BMI values [19,30,32]. However, these hypotheses are based on previously published data and were not directly evaluated in the present study, as information on treatment-related toxicity, dose modifications, or treatment intensity was not available in our dataset. Therefore, these mechanisms should be interpreted as potential explanations rather than conclusions supported by our findings. At the same time, accumulating evidence suggests that body composition may be more informative than BMI alone. In particular, sarcopenia has emerged as a more informative prognostic parameter than BMI in patients treated with CDK4/6 inhibitors [33,34,35], which further questions the biological rationale for grouping underweight and normal-weight patients into a single BMI < 25 kg/m^2^ category.

Although palbociclib, ribociclib, and abemaciclib share a common mechanism of CDK4/6 inhibition, relevant differences in pharmacokinetic properties, kinase selectivity, and toxicity profiles may influence the relationship between BMI and treatment outcomes. Palbociclib and ribociclib are administered intermittently and are more frequently associated with hematologic toxicity, particularly neutropenia, which is dose- and exposure-dependent, whereas abemaciclib is given continuously and demonstrates greater selectivity for CDK4 over CDK6, explaining its lower rates of severe neutropenia but higher gastrointestinal toxicity [36]. In addition, abemaciclib’s higher lipophilicity and tissue penetration, including breast tissue and the blood–brain barrier [37] suggest that adipose tissue distribution could differentially affect its pharmacodynamics compared with palbociclib or ribociclib. As palbociclib was the first CDK4/6 inhibitor approved and reimbursed in Romania (2018), all patients in our cohort received palbociclib. Accordingly, the present findings provide long-term real-world data and should not be generalized to other CDK4/6 inhibitors without caution.

From a biological perspective, adipocytes function not merely as structural elements but as active regulators, secreting metabolic substrates, growth factors, adipokines, and pro-inflammatory cytokines within the tumor microenvironment (TME) [38]. These factors are implicated in promoting tumor proliferation, invasion, angiogenesis, immune evasion, and resistance to systemic therapy [38]. Emerging data indicate that CDK4 and CDK6 are involved in the regulation of adipocyte biology, modulating metabolic pathways related to insulin signaling, adipogenesis, thermogenesis, and lipid metabolism [39,40]. Moreover, CDK4 and CDK6 have been recognized as contributors to the pathogenesis of metabolic disorders, such as diabetes and obesity [39]. Specifically, CDK4 has been implicated in the regulation of adipocyte differentiation and function through activation of peroxisome proliferator-activated receptor γ (*PPAR-γ*), supporting adipogenesis and adipocyte metabolic activity [23]. Furthermore, CDK6 suppresses the white-to-beige adipocyte transition through inhibition of the transcription factor *RUNX1*, thereby emerging as a potential therapeutic target in obesity and associated metabolic disorders [41]. Enhanced lipogenesis in white adipose tissue of mouse models with impaired CDK6 activity supports a role for CDK4/6 inhibition in obesity-related metabolic disorders [42]. Consequently, CDK4/6 inhibitors may modulate diet-induced obesity and influence metabolic outcomes in cancer patients. Cancer-associated adipocytes (CAAs) are key components of the tumor microenvironment, promoting cancer progression through metabolic and paracrine interactions with tumor cells [38]. Palbociclib has been shown to counteract the proliferative effects of cancer-associated adipose tissue (CAAT) secretome on BC cells, which contains factors such as leptin and insulin-like growth factor binding protein 2 (*IGFBP2*) that activate oncogenic pathways including PI3K/AKT and STAT3, promoting tumor growth and therapeutic resistance [43]. In this context, the lack of association observed in our study further supports the view that BMI does not adequately capture the complexity of host–tumor interactions in HR+/HER2− MBC.

Obesity, characterized by chronic low-grade inflammation, creates a pro-inflammatory immune environment that may be modulated by CDK4/6 inhibitors [33]. A recent meta-analysis suggested that patients with MBC and higher BMI may derive greater PFS benefit from CDK4/6 inhibitor therapy, particularly when adiposity is assessed by visceral adipose tissue (VAT) rather than BMI alone [44,45]. In a study by Yucel et al., treatment with CDK4/6 inhibitors was associated with a significant reduction in the VAT index at 6 months (mean change −4.21; *p* = 0.02) [33]. Collectively, these findings support the potential of CDK4/6 inhibitors to disrupt adipose-rich TME interactions and suggest that targeting CDK4/6 may help counteract both obesity-related metabolic dysregulation and cancer progression [46].

Although obesity has been linked to inferior prognosis in EBC, largely attributed to estrogen-dependent tumor biology, its prognostic relevance in the metastatic setting appears more heterogeneous, with some data suggesting a correlation between higher BMI and improved survival outcomes in MBC [47]. Multiple hypotheses have been proposed to account for the observed obesity paradox across different stages of breast cancer. In EBC, sustained hormonal exposure and chronic low-grade inflammatory processes are thought to adversely affect clinical outcomes among obese patients.

Estrogens produced by adipose tissue, together with inflammatory mediators released in response to adipocyte hypoxia, are believed to promote the growth of estrogen receptor-positive BC [9]. In the metastatic setting, where treatment goals are primarily palliative, short-term host-related factors play a more prominent role in shaping prognosis. Excess adipose tissue may act as an energy reserve during systemic therapy, potentially mitigating cancer-related cachexia, which could have a greater impact on survival than the adverse metabolic effects of obesity [48]. In addition, underweight patients may be at increased risk of sarcopenia, a condition consistently associated with poorer clinical outcomes in several studies [33,34].

Our study findings may have important implications for clinical practice, particularly by emphasizing the need for a more individualized approach to treatment beyond conventional metrics such as BMI alone. In our analysis, patients were categorized using a predefined BMI cutoff (≥25 kg/m^2^), selected as an appropriate threshold; however, prior evidence suggests that the choice of BMI cutoffs and categorization methods may significantly influence study results and their interpretation [49], potentially contributing to the absence of an observed association between BMI and PFS or OS in our cohort. Although BMI is widely used, it may not adequately capture the complex interactions between body composition, drug exposure, and treatment-related toxicity in patients receiving CDK4/6 inhibitors. Adopting a more personalized and holistic characterization of patients, beyond BMI alone, may represent an important step toward improving treatment strategies and enhancing clinical outcomes in patients with HR+/HER2− MBC treated with CDK4/6 inhibitors.

These findings should be interpreted in the context of several inherent limitations. First, the retrospective design may introduce selection bias and limit the ability to establish causal relationships between variables. Second, although this analysis was conducted within a national, multicenter real-world cohort, only patients with available baseline height and weight data were included, reducing the sample size for certain analyses. Similarly, in accordance with the original study protocol, only patients who received palbociclib for at least 3 months were included in the analysis, and patients with severe symptomatic visceral disease were excluded. These criteria may have excluded early unfavorable events, such as rapid progression, early death, or early treatment discontinuation, resulting in a cohort enriched for clinically more favorable cases. Third, BMI was used as a surrogate marker of adiposity; however, it does not accurately reflect body composition, as it cannot distinguish between adipose tissue and skeletal muscle mass or capture variations in fat distribution, such as subcutaneous versus visceral adiposity. In addition, reliance on a single baseline BMI measurement does not account for dynamic changes during treatment. Additionally, the use of a dichotomized BMI variable may have oversimplified biologically distinct categories, potentially masking more complex or non-linear relationships between BMI and clinical outcomes. Furthermore, all patients in this cohort received palbociclib, as it was the first CDK4/6 inhibitor reimbursed in Romania, allowing for longer follow-up. Therefore, the findings may not be fully generalizable to other CDK4/6 inhibitors. Finally, although multiple clinicopathological variables were included in the multivariate analyses and efforts were made to adjust for confounding factors, residual confounding cannot be excluded. Important variables, such as lifestyle characteristics, metabolic status, or inflammatory markers, were not available, and potential biases, including reverse causation or cancer-related weight changes, may have influenced the results.

Despite these limitations, this study has several important strengths. It is based on a relatively large, national, multicenter real-world cohort, reflecting routine clinical practice. As a real-world study, it captures variability in treatment administration and patient monitoring compared with controlled clinical trials, including differences in imaging frequency and response assessment, which may introduce heterogeneity in recorded outcomes. This highlights the need for more rigorous documentation and greater standardization of real-world data collection to improve the comparability and reliability of observational research. Moreover, this study specifically addresses the impact of BMI on outcomes in patients treated with CDK4/6 inhibitors, a topic that remains insufficiently explored in the current literature. The consistency of findings across multiple endpoints, including PFS, OS, and response rates, further supports our results and contributes to a better understanding of the complex relationship between body composition and treatment outcomes in MBC.

## 5. Conclusions

Although CDK4/6 inhibitors have significantly improved outcomes in patients with HR+/HER2− MBC, identifying reliable predictors of treatment response remains an unmet clinical need. In this national real-world cohort of patients who tolerated at least three months of palbociclib, baseline BMI categorized using a ≥25 kg/m^2^ threshold was not significantly associated with PFS, OS, or treatment response, and this finding was consistent across treatment lines and analytical approaches.

These findings should be interpreted with caution, given the retrospective design, the selection criteria, and the limitations of BMI as a surrogate marker of body composition. While clinically informative, the results should not be interpreted as definitive evidence that body weight has no influence on palbociclib efficacy. At the same time, they underscore the need for more refined and individualized approaches incorporating detailed assessments of adipose tissue distribution, skeletal muscle mass, and metabolic parameters. Furthermore, the use of a dichotomized BMI threshold (≥25 kg/m^2^) represents a simplified categorization that may obscure biologically relevant differences in body composition.

Future prospective studies integrating comprehensive measures of adiposity, skeletal muscle mass, and metabolic biomarkers are warranted to better elucidate the role of body composition in treatment outcomes among patients receiving CDK4/6 inhibitors. Moving beyond BMI toward a more holistic and personalized characterization of patients with HR+/HER2− MBC may represent an important step in optimizing therapeutic strategies and improving outcomes in this population.

## Figures and Tables

**Table 1:** Clinicopathological characteristics of HR+/HER2− MBC patients stratified by BMI categories (<25 vs. ≥25 kg/m^2^).

Characteristic	Total (*N* = 326)	BMI < 25 *N* = 109 (33.44%)	BMI ≥ 25 *N* = 217 (66.56%)	*p*-Value
Age at diagnosis Median (IQR)	60.0 (49.0–67.0)	54.0 (46.0–66.0)	61.0 (50.0–67.0)	0.039
Menopausal status Premenopausal Postmenopausal	58 (17.79%) 268 (82.21%)	25 (22.94%) 84 (77.06%)	33 (15.21%) 184 (84.79%)	0.085
Hypertension Yes No	122 (37.42%) 204 (62.58%)	30 (27.52%) 79 (72.48%)	92 (42.40%) 125 (57.60%)	0.009
Diabetes Yes No	40 (12.27%) 286 (87.73%)	10 (9.17%) 99 (90.83%)	30 (13.82%) 187 (86.18%)	0.227
BMI Median (IQR)	26.65 (23.70–30.40)	22.60 (21.40–23.70)	28.90 (26.70–32.00)	
Histologic type Ductal Lobular Others	188 (57.67%) 60 (18.40%) 78 (23.93%)	66 (60.55%) 19 (17.43%) 24 (22.02%)	122 (56.22%) 41 (18.89%) 54 (24.88%)	0.752
Nuclear grade G1 G2 G3	56 (17.18%) 224 (68.71%) 46 (14.11%)	17 (15.60%) 81 (74.31%) 11 (10.09%)	39 (17.97%) 143 (65.90%) 35 (16.13%)	0.239
Luminal subtype Luminal A Luminal B	128 (39.26%) 198 (60.74%)	40 (36.70%) 69 (63.30%)	88 (40.55%) 129 (59.45%)	0.501
Ki-67 status <20% ≥20%	116 (37.18%) 196 (62.82%)	41 (39.42%) 63 (60.58%)	75 (36.06%) 133 (63.94%)	0.562
HER2 receptor status Negative Low	173 (52.92%) 153 (47.08%)	59 (54.13%) 50 (45.87%)	114 (52.31%) 103 (47.69%)	0.757
Prior neo/adjuvant endocrine therapy Anastrozol Exemestan Letrozol Tamoxifen Sequential Tamoxifen—AI None	29 (8.90%) 6 (1.84%) 45 (13.80%) 43 (13.19%) 16 (4.91%) 187 (57.36%)	12 (11.01%) 3 (2.75%) 17 (15.60%) 15 (13.76%) 5 (4.59%) 57 (52.29%)	17 (7.83%) 3 (1.38%) 28 (12.90%) 28 (12.90%) 11 (5.07%) 130 (59.91%)	0.909
Prior neo/adjuvant chemotherapy Yes No None	108 (33.13%) 28 (8.59%) 190 (58.28%)	39 (35.78%) 10 (9.17%) 60 (55.05%)	69 (31.80%) 18 (8.29%) 130 (59.91%)	0.969
Pattern of metastatic Presentation De novo Recurrent	184 (56.44%) 142 (43.56%)	57 (52.29%) 52 (47.71%)	127 (58.53%) 90 (41.47%)	0.284
Bone metastases Yes No	215 (65.95%) 111 (34.05%)	70 (64.22%) 39 (35.78%)	145 (66.82%) 72 (33.18%)	0.640
Brain metastases Yes No	12 (3.68%) 314 (96.32%)	3 (2.75%) 106 (97.25%)	9 (4.15%) 208 (95.85%)	0.757
Liver metastases Yes No	62 (19.02%) 264 (80.98%)	24 (22.02%) 85 (77.98%)	38 (17.51%) 179 (82.49%)	0.328
Lung metastases Yes No	123 (37.73%) 203 (62.27%)	38 (34.86%) 71 (65.14%)	85 (39.17%) 132 (60.83%)	0.449
Others metastasis Yes No	153 (46.93%) 173 (53.07%)	51 (46.79%) 58 (53.21%)	102 (47.00%) 115 (53.00%)	0.971
Number of metastatic sites 1 2 3	140 (42.94%) 87(26.69%) 99(30.37%)	44 (40.37%) 32 (29.36%) 33 (30.28%)	96 (44.24%) 55 (25.35%) 66 (30.41%)	0.708
Visceral sites Yes No	177 (54.29%) 149 (45.71%)	61 (55.96%) 48 (44.04%)	116 (53.46%) 101 (46.54%)	0.668
Treatment line of palbociclib I II	229 (70.25%) 97 (29.75%)	77 (70.64%) 32 (29.36%)	152 (70.05%) 65 (29.95%)	0.871
Concurrent endocrine therapy AI Fulvestrant	232 (71.17%) 94 (28.83%)	79 (72.48%) 30 (27.52%)	153 (70.51%) 64 (29.49%)	0.711

Abbreviations: BMI, body mass index; IQR, interquartile range; HER2, human epidermal growth factor receptor 2; AI, aromatase inhibitor.

**Table 2:** Tumor response according to baseline BMI.

	BMI < 25	BMI ≥ 25	*p*-Value
Objective response rate (ORR), n (%) OR (95% CI)	25 (29.07)	52 (28.89)	1.000
	Ref	1.031 (0.580–1.862)	0.918
Best objective response, n (%) Complete response Partial response Stable disease Progressive disease	1 (1.16) 24 (27.91) 53 (61.63)	3 (1.67) 49 (27.22) 107 (59.44)	1.000 1.000 0.790
	8 (9.30)	21 (11.67)	0.676
Clinical benefit rate (CBR), n (%) OR (95% CI)	78 (90.70)	159 (88.33)	0.676
	Ref	0.765 (0.301–1.786)	0.551
Evaluable for response, n (%)	86 (78.9%)	180 (83.0%)	

Abbreviations: BMI, body mass index; ORR, objective response rate; CBR, clinical benefit rate; BOR, best overall response. CBR was defined as CR + PR + SD with duration ≥6 months. Patients with stable disease lasting <6 months were classified as non-responders for the purpose of CBR calculation.

**Table 3:** Univariate and multivariate Cox regression analysis for progression-free survival in patients with HR+/HER2− MBC treated with palbociclib.

Characteristic	Univariate Analysis		Multivariate Analysis	
	HR (95% CI)	*p*	HR (95% CI)	*p*
Age	1.00 (0.99–1.01)	0.656	-	-
Menopausal status Premenopausal Postmenopausal	1.03 (0.77–1.39)	0.840	-	-
			
Hypertension Yes No	0.93 (0.74–1.17)	0.552	-	-
			
Diabetes Yes No	1.30 (0.92–1.83)	0.139	-	-
BMI	0.86 (0.62–1.20)	0.373	-	-
Histologic type Ductal Lobular Others	- 0.75 (0.55–1.02) 1.20 (0.92–1.58)	- 0.063 0.183	- - -	- - -
Nuclear grade G1 G2 G3	- 1.25 (0.93–1.70) 1.35 (0.91–2.02)	- 0.144 0.138	1.09 (0.78–1.52) 1.21 (0.79–1.85)	0.617 0.374
Luminal subtype Luminal A Luminal B	- 1.31 (1.04–1.65)	- 0.021	- -	- -
Ki-67 status <20% ≥20%	0.77 (0.61–0.97) -	0.025 -	0.80 (0.62–1.03) -	0.084
HER2 receptor status Negative Low	- 1.09 (0.87–1.36)	- 0.461	- -	- -
Pattern of metastatic presentation De novo Recurrent	- 1.07 (0.85–1.34)	- 0.575	- -	- -
Bone metastases Yes No	1.13 (0.89–1.43) -	0.319 -	- -	- -
Brain metastases Yes No	1.39 (0.78–2.47) -	0.270 -	1.45 (0.81–2.60) -	0.211 -
Lung metastases Yes No	1.08 (0.86–1.36) -	0.513 -	-	-
Liver metastases Yes No	1.56 (1.17–2.09) -	0.002 -	1.59 (1.19–2.14) -	0.002 -
Others metastasis Yes No	0.98 (0.78–1.22) -	0.839 -	-	-
Number of metastatic sites 1 2 3	- 1.40 (1.06–1.85) 1.35 (1.03–1.76)	- 0.019 0.029	- -	- -
Visceral sites Yes No	1.30 (1.04–1.64) -	0.022 -	-	-
Treatment line of palbociclib I II	- 1.52 (1.19–1.95)	- 0.001	- -	- -

Abbreviations: HR, hazard ratio; CI, confidence interval; BMI, body mass index; HER2, human epidermal growth factor receptor 2; AI, aromatase inhibitor. Hazard ratios were estimated using Cox proportional hazards regression models. Variables included in the multivariate model were selected based on univariate results and clinical relevance.

**Table 4:** Univariate and multivariate Cox regression analysis for overall survival in patients with HR+/HER2− MBC treated with palbociclib.

Characteristic	Univariate Analysis		Multivariate Analysis	
	HR (95% CI)	*p*	HR (95% CI)	*p*
Age	1.00 (0.98–1.02)	0.731	-	-
Menopausal status Premenopausal Postmenopausal	0.70 (0.35–1.40)	0.307	-	-
	-	-		
Hypertension Yes No	1.19 (0.76–1.85)	0.447	-	-
			
Diabetes Yes No	1.23 (0.65–2.34)	0.519	-	-
BMI	0.82 (0.52–1.30)	0.398	-	-
Histologic type Ductal Lobular Others	- 0.66 (0.36–1.20) 0.49 (0.24–0.98)	- 0.172 0.044	- - - -	- - -
Nuclear grade G1 G2 G3	- 2.38 (1.09–5.21) 3.26 (1.33–8.03)	- 0.030 0.010	1.74 (0.76–3.99) 1.21 (0.79–1.85)	0.190 0.087
Luminal subtype Luminal A Luminal B	- 2.09 (1.28–3.42)	- 0.003	- -	- -
Ki-67 status <20% ≥20%	0.49 (0.29–0.81) -	0.006 -	0.59 (0.34–1.00) -	0.051 -
HER2 receptor status Negative Low	- 1.16 (0.75–1.81)	- 0.505	- -	- -
Pattern of metastatic presentation De novo Recurrent	- 1.00 (0.64–1.56)	- 0.989	- -	- -
Bone metastases Yes No	1.25 (0.78–2.00) -	0.364 -	- -	- -
Brain metastases Yes No	2.64 (1.15–6.09) -	0.023 -	2.78 (1.16–6.66) -	0.022 -
Lung metastases Yes No	1.18 (0.75–1.84) -	0.477 -	-	-
Liver metastases Yes No	2.18 (1.33–3.58) -	0.002 -	2.33 (1.40–3.88) -	0.001 -
Others metastasis Yes No	1.12 (0.72–1.74) -	0.611 -	-	-
Number of metastatic sites 1 2 3	- 1.61 (0.93–2.80) 1.71 (1.02–2.89)	- 0.089 0.043	- -	- -
Visceral sites Yes No	1.95 (1.22–3.10) -	0.005 -	-	-
Treatment line of palbociclib I II	- 1.40 (0.86–2.28)	- 0.173	- -	- -

Abbreviations: HR, hazard ratio; CI, confidence interval; BMI, body mass index; HER2, human epidermal growth factor receptor 2; AI, aromatase inhibitor. Hazard ratios were estimated using Cox proportional hazards regression models. Variables included in the multivariate model were selected based on univariate results and clinical relevance.

## Data Availability

The data generated or analyzed during this study are included in this published article or are available from the corresponding author on reasonable request.

## Abbreviations

The following abbreviations are used in this manuscript:

BMI	Body mass index
WHO	World Health Organization
BC	Breast cancer
EBC	Early breast cancer
HR+	Hormone receptor-positive
NCCN	National Comprehensive Cancer Network
MBC	Metastatic breast cancer
HER2−	Human epidermal growth factor receptor 2–negative
FISH	Fluorescence in situ hybridization
ISH	In situ hybridization
ORR	Objective response rate
CBR	Clinical benefit rate
CR	Complete response
PR	Partial response
SD	Stable disease
RECIST	Response Evaluation Criteria in Solid Tumors
CT	Computed tomography
HR	Hazard ratio
CI	Confidence interval
OR	Odds ratio
AI	Aromatase inhibitor
TME	Tumor microenvironment
PPAR-γ	Peroxisome proliferator-activated receptor γ
CAAs	Cancer-associated adipocytes
CAAT	Cancer-associated adipose tissue
IGFBP2	Insulin-like growth factor binding protein 2
VAT	Visceral adipose tissue

## References

[1] Yu L., Yuan J., Meng M., Pan H., Ge L., Wang L., Li X. (2026). Unravelling the Obesity Paradox in Cancer: An Umbrella Review of Protective Associations and Evidence Credibility across 13 Malignancies. Metab.-Clin. Exp..

[2] Rubino F., Cummings D.E., Eckel R.H., Cohen R.V., Wilding J.P.H., Brown W.A., Stanford F.C., Batterham R.L., Farooqi I.S., Farpour-Lambert N.J. (2025). Definition and Diagnostic Criteria of Clinical Obesity. Lancet Diabetes Endocrinol..

[3] World Health Organization Obesity and Overweight. https://www.who.int/news-room/fact-sheets/detail/obesity-and-overweight.

[4] Phelps N.H., Singleton R.K., Zhou B., Heap R.A., Mishra A., Bennett J.E., Paciorek C.J., Lhoste V.P., Carrillo-Larco R.M., Stevens G.A. (2024). Worldwide Trends in Underweight and Obesity from 1990 to 2022: A Pooled Analysis of 3663 Population-Representative Studies with 222 Million Children, Adolescents, and Adults. Lancet.

[5] Ecancer Breast Cancer Incidence Is Rising Globally, but Mortality Disproportionately Affects the Global South. http://ecancer.org/en/news/26289-breast-cancer-incidence-is-rising-globally-but-mortality-disproportionately-affects-the-global-south.

[6] Wen H., Deng G., Shi X., Liu Z., Lin A., Cheng Q., Zhang J., Luo P. (2024). Body Mass Index, Weight Change, and Cancer Prognosis: A Meta-Analysis and Systematic Review of 73 Cohort Studies. ESMO Open.

[7] Dauccia C., Bruzzone M., Arecco L., Blondeaux E., Sirico M., Gerosa R., Franzoi M.A., Brandão M., Perrone L., Pedrazzoli P. (2026). Association between Body Mass Index and Risk of Breast Cancer According to Breast Cancer Subtypes: A Systematic Review and Meta-Analysis. Breast.

[8] Chan D.S.M., Vieira A.R., Aune D., Bandera E.V., Greenwood D.C., McTiernan A., Navarro Rosenblatt D., Thune I., Vieira R., Norat T. (2014). Body Mass Index and Survival in Women with Breast Cancer—Systematic Literature Review and Meta-Analysis of 82 Follow-up Studies. Ann. Oncol..

[9] Brown K.A. (2021). Metabolic Pathways in Obesity-Related Breast Cancer. Nat. Rev. Endocrinol..

[10] Guidelines Detail. https://www.nccn.org/guidelines/guidelines-detail?category=1&id=1419.

[11] Gevorgyan A., Bregni G., Galli G., Ganzinelli M., Martinetti A., Lo Vullo S., Mariani L., Festinese F., Sottotetti E., de Braud F. (2016). Body Mass Index and Clinical Benefit of Fulvestrant in Postmenopausal Women with Advanced Breast Cancer. Tumori J..

[12] Martel S., Poletto E., Ferreira A.R., Lambertini M., Sottotetti F., Bertolini I., Montemurro F., Bernardo A., Risi E., Zanardi E. (2018). Impact of Body Mass Index on the Clinical Outcomes of Patients with HER2-Positive Metastatic Breast Cancer. Breast.

[13] Lammers S.W.M., Thurisch H., Vriens I.J.H., Meegdes M., Engelen S.M.E., Erdkamp F.L.G., Dercksen M.W., Vriens B.E.P.J., Aaldering K.N.A., Pepels M.J.A.E. (2024). The Prognostic Impact of BMI in Patients with HR+/HER2− Advanced Breast Cancer: A Study of the SONABRE Registry. Breast Cancer Res. Treat..

[14] Zewenghiel L., Lindman H., Valachis A. (2018). Impact of Body Mass Index on the Efficacy of Endocrine Therapy in Patients with Metastatic Breast Cancer—A Retrospective Two-Center Cohort Study. Breast.

[15] Petrelli F., Cortellini A., Indini A., Tomasello G., Ghidini M., Nigro O., Salati M., Dottorini L., Iaculli A., Varricchio A. (2021). Association of Obesity with Survival Outcomes in Patients with Cancer. JAMA Netw. Open.

[16] Jung Y.B., Ahn H.K., Shin H.-Y., Hong J.H., Rim C.H. (2026). The Impact of Obesity on Treatment Outcomes in Patients with Hormone Receptor–Positive HER2-Negative Metastatic Breast Cancer Receiving CDK 4/6 Inhibitors. Cancer Res. Treat..

[17] Female Breast Cancer Subtypes—Cancer Stat Facts. https://seer.cancer.gov/statfacts/html/breast-subtypes.html.

[18] Morrison L., Loibl S., Turner N.C. (2024). The CDK4/6 Inhibitor Revolution—A Game-Changing Era for Breast Cancer Treatment. Nat. Rev. Clin. Oncol..

[19] Franzoi M.A., Eiger D., Ameye L., Ponde N., Caparica R., De Angelis C., Brandão M., Desmedt C., Di Cosimo S., Kotecki N. (2021). Clinical Implications of Body Mass Index in Metastatic Breast Cancer Patients Treated with Abemaciclib and Endocrine Therapy. JNCI J. Natl. Cancer Inst..

[20] Çağlayan D., Koçak M.Z., Geredeli Ç., Atcı M.M., Tatlı A.M., Göksu S.S., Eryılmaz M.K., Araz M., Artaç M. (2024). The Impact of Body Mass Index on the Progression-Free Survival of CDK 4/6 Inhibitors in Metastatic Breast Cancer Patients. Future Oncol..

[21] Fasching P.A., Decker T., Hartkopf A., Nusch A., Heinrich B.J., Kurbacher C., Fuchs R., Tesch H., Krabisch P., Huober J. (2024). Efficacy, Safety, and Prognosis Prediction in Patients Treated with Ribociclib in Combination with Letrozole: Final Results of Phase 3b RIBECCA Study in Hormone Receptor Positive, Human Epidermal Growth Factor Receptor-2 Negative, Locally Advanced or Metastatic Breast Cancer. Eur. J. Cancer.

[22] Knudsen E.S., Schultz E., Hamilton D., Attwood K., Edge S., O’Connor T., Levine E., Witkiewicz A.K. (2022). Real-World Experience with CDK4/6 Inhibitors for Metastatic HR+/HER2− Breast Cancer at a Single Cancer Center. Oncologist.

[23] Lopez-Mejia I.C., Castillo-Armengol J., Lagarrigue S., Fajas L. (2017). Role of Cell Cycle Regulators in Adipose Tissue and Whole Body Energy Homeostasis. Cell. Mol. Life Sci..

[24] Oprean C.M., Badau L.M., Petrita R., Median M.D., Dema A. (2025). Real-World, National Study of Palbociclib in HR+/HER2− Metastatic Breast Cancer: A 2.5-Year Follow-Up PALBO01/2021. Diagnostics.

[25] World Health Organization Obesity. https://www.who.int/health-topics/obesity.

[26] Zhang F., Mesias J.A. (2024). Abstract PO3-05-09: The Impact of Body Mass Index on CDK4/6 Inhibitor Treatment Survival Outcomes in Metastatic Breast Cancer. Cancer Res..

[27] Karahan L., Akyildiz A., Sahin T.K., Batu M.A., Ersan C., Onur M.R., Aksoy S., Guven D.C. (2026). Prognostic Value of Baseline Sarcopenia and Adipose Tissue Indices in HR+/HER2− Metastatic Breast Cancer Treated with CDK4/6 Inhibitors: A Retrospective Cohort Study. J. Clin. Med..

[28] Chen B.-F., Tsai Y.-F., Chao T.-C., Lien P.-J., Lin Y.-S., Feng C.-J., Chen Y.-J., Cheng H.-F., Liu C.-Y., Lai J.-I. (2024). Real-World Experience with CDK4/6 Inhibitors in Hormone Receptor-Positive Metastatic and Recurrent Breast Cancer: Findings from an Asian Population. Clin. Exp. Med..

[29] Badau L.M., Oprean C.M., Ciocoiu A.D., Epure P., Vlaicu B. (2026). The Prognostic and Predictive Value of Body Mass Index in Patients with HR+/HER2− Breast Cancer Treated with CDK4/6 Inhibitors: A Systematic Literature Review. Cancers.

[30] Roncato R., Peruzzi E., Gerratana L., Posocco B., Nuzzo S., Montico M., Orleni M., Corsetti S., Bartoletti M., Gagno S. (2023). Clinical Impact of Body Mass Index on Palbociclib Treatment Outcomes and Effect on Exposure. Biomed. Pharmacother..

[31] Roncato R., Angelini J., Pani A., Cecchin E., Sartore-Bianchi A., Siena S., De Mattia E., Scaglione F., Toffoli G. (2020). CDK4/6 Inhibitors in Breast Cancer Treatment: Potential Interactions with Drug, Gene, and Pathophysiological Conditions. Int. J. Mol. Sci..

[32] Iwata H., Im S.-A., Masuda N., Im Y.-H., Inoue K., Rai Y., Nakamura R., Kim J.H., Hoffman J.T., Zhang K. (2017). PALOMA-3: Phase III Trial of Fulvestrant with or Without Palbociclib in Premenopausal and Postmenopausal Women with Hormone Receptor-Positive, Human Epidermal Growth Factor Receptor 2-Negative Metastatic Breast Cancer That Progressed on Prior Endocrine Therapy-Safety and Efficacy in Asian Patients. J. Glob. Oncol..

[33] Yücel K.B., Aydos U., Sütcüoglu O., Kılıç A.C.K., Özdemir N., Özet A., Yazıcı O. (2024). Visceral Obesity and Sarcopenia as Predictors of Efficacy and Hematological Toxicity in Patients with Metastatic Breast Cancer Treated with CDK 4/6 Inhibitors. Cancer Chemother. Pharmacol..

[34] Kripa E., Rizzo V., Galati F., Moffa G., Cicciarelli F., Catalano C., Pediconi F. (2022). Do Body Composition Parameters Correlate with Response to Targeted Therapy in ER+/HER2− Metastatic Breast Cancer Patients? Role of Sarcopenia and Obesity. Front. Oncol..

[35] Franzoi M.A., Vandeputte C., Eiger D., Caparica R., Brandão M., De Angelis C., Hendlisz A., Awada A., Piccart M., de Azambuja E. (2020). Computed Tomography-Based Analyses of Baseline Body Composition Parameters and Changes in Breast Cancer Patients under Treatment with CDK 4/6 Inhibitors. Breast Cancer Res. Treat..

[36] Marra A., Curigliano G. (2019). Are All Cyclin-Dependent Kinases 4/6 Inhibitors Created Equal?. npj Breast Cancer.

[37] Braal C.L., Jongbloed E.M., Wilting S.M., Mathijssen R.H.J., Koolen S.L.W., Jager A. (2021). Inhibiting CDK4/6 in Breast Cancer with Palbociclib, Ribociclib, and Abemaciclib: Similarities and Differences. Drugs.

[38] Mukherjee A., Bilecz A.J., Lengyel E. (2022). The Adipocyte Microenvironment and Cancer. Cancer Metastasis Rev..

[39] Pellarin I., Dall’Acqua A., Favero A., Segatto I., Rossi V., Crestan N., Karimbayli J., Belletti B., Baldassarre G. (2025). Cyclin-Dependent Protein Kinases and Cell Cycle Regulation in Biology and Disease. Signal Transduct. Target. Ther..

[40] Hou X., Zhang Y., Li W., Hu A.J., Luo C., Zhou W., Hu J.K., Daniele S.G., Wang J., Sheng J. (2018). CDK6 Inhibits White to Beige Fat Transition by Suppressing RUNX1. Nat. Commun..

[41] Hu A.J., Li W., Pathak A., Hu G.-F., Hou X., Farmer S.R., Hu M.G. (2023). CDK6 Is Essential for Mesenchymal Stem Cell Proliferation and Adipocyte Differentiation. Front. Mol. Biosci..

[42] Hu A.J., Li W., Dinh C., Zhang Y., Hu J.K., Daniele S.G., Hou X., Yang Z., Asara J.M., Hu G.-F. (2024). CDK6 Inhibits de Novo Lipogenesis in White Adipose Tissues but Not in the Liver. Nat. Commun..

[43] Lore L., An H., Evelyne L., Mieke V.B., Jo V., Dawn M., Geert B., Rudy V.D.B., Cathérine M., Marc B. (2017). Secretome Analysis of Breast Cancer-Associated Adipose Tissue to Identify Paracrine Regulators of Breast Cancer Growth. Oncotarget.

[44] Lappano R., Rigiracciolo D.C., Belfiore A., Maggiolini M., De Francesco E.M. (2020). Cancer Associated Fibroblasts: Role in Breast Cancer and Potential as Therapeutic Targets. Expert Opin. Ther. Targets.

[45] Hu D., Li Z., Zheng B., Lin X., Pan Y., Gong P., Zhuo W., Hu Y., Chen C., Chen L. (2022). Cancer-associated Fibroblasts in Breast Cancer: Challenges and Opportunities. Cancer Commun..

[46] Scordamaglia D., Talia M., Zicarelli A., Mondino A.A., De Rosis S., Di Dio M., Silvestri F., Meliti C., Cirillo F., De Francesco E.M. (2025). Alternate Actions of CDK4/6 Inhibitors beyond Cell Cycle Blockade: Unexplored Roles in Therapy Resistance. Cancer Metastasis Rev..

[47] Alarfi H., Salamoon M., Kadri M., Alammar M., Haykal M.A., Alseoudi A., Youssef L.A. (2017). The Impact of Baseline Body Mass Index on Clinical Outcomes in Metastatic Breast Cancer: A Prospective Study. BMC Res. Notes.

[48] Gallo M., Adinolfi V., Barucca V., Prinzi N., Renzelli V., Barrea L., Di Giacinto P., Ruggeri R.M., Sesti F., Arvat E. (2021). Expected and Paradoxical Effects of Obesity on Cancer Treatment Response. Rev. Endocr. Metab. Disord..

[49] Wu Y., Li D., Vermund S.H. (2024). Advantages and Limitations of the Body Mass Index (BMI) to Assess Adult Obesity. Int. J. Environ. Res. Public Health.

